# Association analysis of genetic polymorphisms of factor V, factor VII and fibrinogen β chain genes with human abdominal aortic aneurysm

**DOI:** 10.3892/etm.2012.608

**Published:** 2012-06-12

**Authors:** KATARZYNA OSZAJCA, KONRAD WROŃSKI, GRAŻYNA JANISZEWSKA, MAŁGORZATA BIEŃKIEWICZ, MICHAŁ PANEK, JACEK BARTKOWIAK, JANUSZ SZEMRAJ

**Affiliations:** 1 Department of Medical Biochemistry, Medical University of Lodz, Lodz 90-419;; 2 General and Vascular Surgery Department, M. Pirogow Regional Specialist Hospital, Lodz 90-531;; 3 Department of Quality Control and Radiological Protection, Medical University of Lodz, Lodz 92-216;; 4 Department of Internal Diseases, Asthma and Allergy, Medical University of Lodz, Lodz 90-153, Poland

**Keywords:** abdominal aortic aneurysm, factor V 1691 G/A polymorphism, factor VII −323 0/10 bp polymorphism, fibrinogen β-chain −455 G/A polymorphism, restriction fragment length polymorphism

## Abstract

Increased activity of the coagulation system is associated with the increased risk of many arterial thrombotic diseases and atherosclerosis. The purpose of this study was to evaluate the influence of selected polymorphisms in genes coding for coagulation factor V (1691 G/A, the so-called Leiden mutation), factor VII (−323 0/10 bp insertion/deletion) and fibrinogen β chain (−455 G/A) on the risk of abdominal aortic aneurysm, a particular form of atherothrombosis. We conducted a case-control study of 153 Polish patients hospitalized due to abdominal aortic aneurysm (AAA) and compared the results to those obtained from matched healthy control subjects. The polymorphisms were ascertained through genotyping by polymerase chain reaction and restriction digestion of amplified fragments. The study revealed that individuals carrying heterozygous genotype GA for the fibrinogen β chain −455 G/A mutation had at least a 2-fold greater likelihood of AAA development compared to control subjects (OR=3.01; 95% CI 1.83–4.96). The cases possessing homozygous mutant genotype (AA) had no significant risk of developing AAA compared to the control subjects (OR=1.12; 95% CI 0.33–2.44; p=0.83). Concerning factor V 1691 G/A and factor VII −323 0/10 bp mutations, we did not find any statistically significant correlation between them and AAA occurrence. In conclusion, we suggest that the −455G/A polymorphism of the fibrinogen β chain gene is a potential genetic marker to identify the risk of AAA.

## Introduction

Abdominal aortic aneurysm (AAA) is a particular form of atherothrombosis characterized by a segmental weakening and dilation of the aortic wall and occurs predominantly among men over 65 years of age. In women, AAA is more rare, but represents a higher relative mortality than in men (1). The pathogenesis of AAA has not yet been completely defined, however, certain characteristics and risk factors are currently known. The destruction of the arterial wall may occur due to the extracellular matrix breakdown that results from an imbalance between proteolytic enzymes (particularly metalloproteinases) and their inhibitors (2,3). The literature data also show that thrombotic factors may participate in the development of aortic aneurysms. On this basis, we examined the occurrence of selected polymorphisms in genes coding for factor V (1691 G/A, the so-called Leiden mutation) and VII (−323 0/10 bp insertion/deletion) as well as fibrinogen β chain (−455 G/A) in patients suffering from AAA.

Factor V (FV, also named proaccelerin or labile factor) is an important component of the coagulation cascade, which – in association with factor Xa (the active form of X) – activates prothrombin to thrombin. The mutation resulting in a G→A substitution at nucleotide 1691 (rs6025) produces a missense mutation that substitutes arginine for glutamine at amino acid residue 506 (Arg506Gln; R506Q) in the factor V heavy chain protein product. This sequence variation has also been referred to as the FV Leiden mutation (FVL). The R506Q substitution in *FVL* involves one of the three sites on factor Va that is cleaved by activated protein C (APC). The mutated protein retains normal procoagulant activity, but is less prone to inactivation by APC and results in the susceptibility to a hypercoagulable state (APC resistance, APCR) (4,5). The decreased rate of APC-mediated degradation of FVa cases increases thrombin formation, which is one of the risk factors for the development of thrombosis (6). APCR based on factor V-Leiden mutation is the most common risk factor of inherited thrombophilia (7,8).

Factor VII (FVII) is a vitamin K-dependent protease that plays a relevant role in the extrinsic pathway of blood coagulation. The active form of FVII (FVIIa) activates factors IX and X, thereby initiating the generation of thrombin and fibrin clot formation (9). It is known that a decanucleotide insertion at position −323 (−323 0/10 bp) in the promoter of the *FVII* gene is associated with lower levels of coagulant activity and antigen levels of factor VII (10,11), which may confer protection against thrombosis.

Fibrinogen is the last factor in the clotting cascade. Structurally, it consists of three pairs of polypeptide chains: α, β and γ, which are encoded by three different genes clustered on chromosome 4q23–32 (12). Elevated fibrinogen levels indicate increased hemostatic system activation and represent the primary risk factor in thrombotic disorders. The fibrinogen β chain gene −455 G/A polymorphism (rs1800790) is particularly involved in the rate-limiting steps of the formation of the β chain and is closely related to the elevation of the plasma fibrinogen level (13–15).

## Materials and methods

### Patients

The study population was comprised of 153 Polish patients with AAA, who were admitted to the M. Pirogow Regional Specialist Hospital in Lodz for emergency repair of ruptured AAA or for elective surgery. AAA was defined as an infrarenal aortic diameter of at least 3 cm. The control group consisted of 152 healthy volunteers who had no history or symptoms of aneurysms. Both study and control subjects had no history of other cardiovascular diseases. All participants provided written informed consent to participate.

### DNA isolation and genotyping

Venous blood samples were taken from all study participants. Genomic DNA was isolated from leukocytes obtained from whole blood samples using phenol-chloroform extraction method or QIAamp DNA Blood Mini kit (Qiagen, Hilden, Germany).

All polymorphisms were genotyped using polymerase chain reaction-restriction fragment length polymorphism sites (PCR-RFLP). For PCR-amplification of the polymorphic regions of *FV*, *FVII* and *FGB*, the following primers were used: 5′ GGAACAACACCATGATCAGAGCA 3′ and 5′ TACCCAGGAGACCTAACATGTTC 3′ for FV, 5′ GAGCGGACGGTTTTGTTGCCAGCG 3′ and 5′ GGCCTGGTCTGGAGGCTCTCTTC 3′ for FVII, 5′ AGAATTTGGGAATGCAATCTCTGCTACCT 3′ and 5′ TCCTCATTGTCGTTGACACCTTGGGAC 3′ for FGB. PCR reactions were performed in reaction mixture containing 2 μl of 10X PCR buffer (with 15 mM MgCl_2_), 2 μl of 2.5 mM dNTPs, 100 pmol of each primer, 1 unit of *Taq* DNA polymerase (Fermentas, Qiagen) and 2 μl of genomic DNA and deionized water added to a final volume of 20 μl. Moreover, in the case of FGB −455 G/A polymorphisms, an additional 2 μl of 25 mM MgCl_2_ was used. The amplification reactions were carried out as follows: denaturation phase at 94°C for 5 min, followed by 35 cycles consisting of DNA denaturation at 95°C for 30 sec, the annealing of primers at 58°C (*FV* and *FVII*) or at 60°C (*FGB*) for 30 sec, and the DNA extension at 72°C for 30 sec (*FV* and *FVII*) or for 1 min (*FGB*). The final extension step was performed at 72°C for 10 min. Amplification and correct sizes of the PCR products were confirmed on polyacrylamide gels in Tris/acetate/EDTA buffer in comparison to 100-bp or 1-kb DNA Ladder (Fermentas). After staining with ethidium bromide the gels were photographed under UV light using Image Master VDS system (Pharmacia Biotech).

Ten microliters of the appropriate PCR product was then digested overnight with an excess of proper restriction enzyme: *Mnl*I for *FV* 1691 G/A, *Sty*I for *FVII* −323 0/10 bp and *Hae*III for *FGB* −455 G/A polymorphism, under conditions recommended by the supplier (Fermentas). The digestion products were separated by electrophoresis on polyacrylamide gels, stained and visualized as described above. Molecular sizes of the restriction fragments are shown in Table I.

To confirm the results obtained using PCR-RFLP, selected samples (every tenth) were subjected to DNA sequence analysis carried out by Genomed S.A. (Warsaw, Poland).

### Statistical analysis

The results are reported as percentages (%) or means with standard deviations (± SD). Analysis of association between genotype/allele distribution and AAA risk was performed with the use of the Chi-square test and the 95% confidence intervals (CI) for disease odds ratio (OR) calculated with the use of logistic regression. All the analyses were derived by means of the Statistica v8.0 software. The level of significance was set at p<0.05.

## Results

The study group consisted of 51 females (33.3%) and 102 males (66.6%), with an age range from 44 to 73 years. The mean age in the study population was 59 years (SD=7). The control group consisted of 152 healthy volunteers: 60 females (39.5%) and 92 males (60.5%), with ages ranging from 37 to 70 years (mean 55, SD ± 9). The control subjects had no history or symptoms of aneurysms and other cardiovascular diseases. The cases and controls were homogeneous in gender and age distribution (p>0.20).

The frequencies of the 1691 G/A polymorphism of factor V gene in AAA patients were obtained as follows: wild-type homozygote (GG), 92.7%; heterozygote (GA), 7.3%, while these frequencies in the control group were 98.7 and 1.3%, respectively (Table II). The statistical analysis showed a significant difference in the occurrence of the GA genotype between the cases and controls (OR=5.9; p=0.023), nevertheless, the data were not convincing due to the fact that the 95% confidence intervals (95% CI) showed a wide range of values (1.28–27.5), probably resulting from the small number of GA genotype cases.

For the AAA group tested for the −323 0/10 bp polymorphism of the factor VII gene, we did not observe any significantly higher frequencies of mutant genotypes in comparison to the control population (χ^2^=0.80; df=1; p=0.37). In the case group, 80.3% of subjects were homozygous wild-type (0/0) and 19.7% were heterozygous (0/10). In the control subjects, there were 84.2% carriers of the 0/0 genotype and 15.8% carriers of the 0/10 genotype (Table III).

Furthermore, neither in the case nor in the control group did we find any subjects with the homozygous mutant geno-type for *FV* 1691 G/A and *FVII* −323 0/10 bp polymorphisms, which suggests that these combinations are rare in the Polish population.

Regarding the case of the −455 G/A polymorphism of the fibrinogen β chain gene, we demonstrated significant statistical differences in genotype and allele frequency between AAA patients and controls (χ^2^=20.5; df=2; p=0.00004) (Table IV). The obtained value of the odds ratio for the GA genotype (OR=3.01; 95% CI 1.83–4.96) suggests that the presence of the heterozygote increases at least by 2-fold (but on average by 3-fold) the risk for the development of AAA, and the result achieved high statistical significance (p=0.00002). In contrast, we found that cases possessing the homozygous mutant genotype (AA) had no significant risk for developing AAA compared to the control subjects (OR=1.12; 95% CI 0.33–2.44; p=0.83).

## Discussion

Disturbances of hemostasis are important risk factors of many arterial vascular diseases, involving aortic aneurysms (16–18). The most common form of aortic aneurysm is aneurysm of the abdominal aorta, comprising 75% of cases. The aim of the present study was an attempt to discern a correlation between genetic polymorphisms of selected coagulation genes and occurrence of AAA.

To our knowledge, here, we investigated the −455 G/A polymorphism of the fibrinogen β chain gene promoter as a risk factor for AAA for the first time. This study revealed that individuals carrying the GA genotype had at least a 2-fold greater likelihood of AAA development. Fibrinogen is one of the primary agents in blood clot formation at sites of vascular damage. There is literature data linking *FGB* −455 G/A genotypes to vascular diseases (19–22). On the contrary to our results, these studies report basically a more significant relationship between the polymorphism and the disease in the case of AA compared to the GA genotype. However, in comparison to our study, this discrepancy may be due to a relatively small number of AA genotypes, both in the case and control groups. Many authors have confirmed that individuals carrying the A allele (particularly the AA genotype) have a higher level of plasma fibrinogen (20,23,24), which could be responsible for the development of atherosclerosis and thrombotic diseases (25,26). However, Rallidis *et al* (27) did not find a significant difference in plasma fibrinogen levels between the carriers of the −455 A allele and those homozygous for the −455 G allele. The possible mechanism responsible for the elevated concentration of circulating fibrinogen in A allele carriers was explained by Brown and Fuller (28). They showed that specific binding of a repressor nuclear protein in the region of −455 G/A polymorphism is greatly enhanced when at the −455 site guanine is present. When guanine is replaced by adenine, the binding of this nuclear protein is weaker and in turn the expression of the fibrinogen β chain is not inhibited. The synthesis of the β chain is the rate-limiting step in the formation of mature fibrinogen, and overexpression of *FGB* results in the upregulation of genes coding for the remaining two chains.

Although no data exist concerning the contribution of the −455 G/A polymorphism in the development of AAA, numerous studies have demonstrated an elevated concentration of fibrinogen in AAA patients (18,29–32). Thus, our results may to some extent explain the association between this polymorphism and AAA appearance. However, the exact underlying molecular mechanism of this affect remains to be elucidated.

We also investigated the occurrence of the non-synonymous 1691 G/A mutation in the coagulation factor V gene in AAA patients. We obtained a higher frequency of the 10-bp allele in the AAA patients vs. the controls and the results reached statistical difference. However, in our opinion, the effect is rather indecisive and requires further verification in a larger group of subjects. The appearance of the Leiden mutation in AAA individuals has not yet been well studied. There is only one report showing, contrary to our findings, that AAA is not affected by the factor V 1691 G/A polymorphism (33). What is more, the authors of this study documented an even higher frequency of individuals carrying the A allele among healthy subjects than among AAA patients (4.6 vs. 3.2%, respectively). Although the *FV* 1691 G/A mutation was previously described as a candidate for the increased risk of venous thromboembolism (34,35), its role in arterial thrombotic disease and atherosclerosis remains unclear (36–40).

There are conflicting literature data concerning the influence of the factor VII 0/10 bp polymorphism on factor VII plasma levels. Most of the data have shown that the insertion of a decanucleotide fragment at position −323 in the promoter region of *FVII* is related to lower coagulation activity and antigen level of factor VII (10,41,42). On the other hand, Mtiraoui *et al* (43) did not find such correlation in a population of healthy Tunisians. The elevated activity of factor VII may indicate a tendency for thrombosis, thus suggesting that a decreased level of circulating factor VII may exert a protective effect on their development. However, we did not observe any increased prevalence of the 10 bp allele in the control group vs. AAA patients. It is difficult to discuss these results since, to date, there are no literature data regarding this issue.

To conclude, we suggest that the −455G/A polymorphism of the fibrinogen β chain gene is a potential genetic marker for the identification of the risk of AAA appearance and therefore may form the basis for future investigation in this field.

## Figures and Tables

**Table I:** Restriction enzyme digestion patterns of PCR-amplified DNA.

Polymorphism	Molecular sizes of restriction fragments (bp)		
	Wild-type homozygote	Heterozygote	Mutant homozygote
*FV* 1691 G/A	157, 93, 37	157, 130, 93, 37	157, 130
*FVII* −323 0/10 bp	214	214, 136, 88	136, 88
*FGB* −455 G/A	575, 383, 343	958, 575, 383, 343	958, 343

**Table II:** Distribution of frequencies of genotypes and alleles of factor V gene 1691 G/A polymorphism in AAA patients and controls.

	Cases (n=151) n (%)	Controls (n=152) n (%)	OR (95% CI)	p-value
Genotype				
GG	140 (92.7)	150 (98.7)	0.17 (0.04–0.78)	0.023
GA	11 (7.3)	2 (1.3)	5.89 (1.28–27.5)	
χ^2^=6.57; df=1; p=0.010				
Allele				
G	291 (96.4)	302 (99.3)	0.18 (0.04–0.80)	0.024
A	11 (3.6)	2 (0.7)	5.71 (1.25–26.5)	

**Table III:** Distribution of frequencies of genotypes and alleles of factor VII gene −323 0/10 bp polymorphism in AAA patients and controls.

	Cases (n=152) n (%)	Controls (n=152) n (%)	OR (95% CI)	p-value
Genotype				
0/0 bp	122 (80.3)	128 (84.2)	0.76 (0.40–1.38)	0.37
0/10 bp	30 (19.7)	24 (15.8)	1.31 (0.72–2.37)	
χ^2^=0.80; df=1; p=0.37				
Allele				
0	274 (90.1)	280 (92.1)	0.78 (0.44–1.38)	0.40
10	30 (9.9)	24 (7.9)	1.28 (0.73–2.24)	

**Table IV:** Distribution of frequencies of genotypes and alleles of fibrinogen β chain gene −455 G/A polymorphism in AAA patients and controls.

	Cases (n=134) n (%)	Controls (n=136) n (%)	OR (95% CI)	p-value
Genotype				
GG	36 (26.9)	72 (52.9)	0.33 (0.20–0.55)	0.00001
GA	90 (67.1)	55 (40.5)	3.01 (1.83–4.96)	0.00002
AA	8 (6.0)	9 (6.6)	1.12 (0.33–2.44)	0.83000
χ^2^=20.5; df=2; p=0.00004				
Allele				
G	162 (60.5)	199 (73.2)	0.99 (0.70–1.40)	0.00200
A	106 (39.5)	73 (26.8)	1.78 (1.24–2.57)	
